# Unleashing the potential of cell painting assays for compound activities and hazards prediction

**DOI:** 10.3389/ftox.2024.1401036

**Published:** 2024-07-17

**Authors:** Floriane Odje, David Meijer, Elena von Coburg, Justin J. J. van der Hooft, Sebastian Dunst, Marnix H. Medema, Andrea Volkamer

**Affiliations:** ^1^ Data Driven Drug Design, Center for Bioinformatics, Saarland University, Saarbrücken, Germany; ^2^ Bioinformatics Group, Wageningen University, Wageningen, Netherlands; ^3^ Department Experimental Toxicology and ZEBET, German Federal Institute for Risk Assessment (BfR), German Centre for the Protection of Laboratory Animals (Bf3R), Berlin, Germany

**Keywords:** cell painting assay, morphological profiling, drug development, high-throughput screening, mode of action, bio-activities predictions

## Abstract

The cell painting (CP) assay has emerged as a potent imaging-based high-throughput phenotypic profiling (HTPP) tool that provides comprehensive input data for *in silico* prediction of compound activities and potential hazards in drug discovery and toxicology. CP enables the rapid, multiplexed investigation of various molecular mechanisms for thousands of compounds at the single-cell level. The resulting large volumes of image data provide great opportunities but also pose challenges to image and data analysis routines as well as property prediction models. This review addresses the integration of CP-based phenotypic data together with or in substitute of structural information from compounds into machine (ML) and deep learning (DL) models to predict compound activities for various human-relevant disease endpoints and to identify the underlying modes-of-action (MoA) while avoiding unnecessary animal testing. The successful application of CP in combination with powerful ML/DL models promises further advances in understanding compound responses of cells guiding therapeutic development and risk assessment. Therefore, this review highlights the importance of unlocking the potential of CP assays when combined with molecular fingerprints for compound evaluation and discusses the current challenges that are associated with this approach.

## 1 Introduction

In the field of drug discovery and toxicology, computational and experimental methodologies are closely intertwined for the rational design of compounds and the assessment of their potential hazardous properties. As a result of increasing volumes of data becoming available, computational methods have become an integral part of the compound assessment process, as described by Muratov et al. (Muratov et al., 2020), helping to (de)prioritize compounds to be tested in the wet lab. This can be done by leveraging information from datasets of already tested compounds for the evaluation of yet untested compounds. In drug design, computational methods aid in the faster identification of promising compounds that exhibit the desired bio-activity, thereby accelerating the drug discovery process. Concerning toxicology, the methods can help to flag hazardous compounds (e.g., compounds that harm human cells) early on, which can then be excluded from further testing. In both scenarios, the aim is to reduce the number of compounds for animal testing, and thus, contribute to the 3Rs introduced by Russell and Burch (Russell and Burch, 1959): replacement, reduction, and refinement of animal testing. Classically, the computational assessment of compound bio-activity and toxicity has been relying heavily on structural and physico-chemical information on compounds, utilizing molecular fingerprints to predict molecular properties or toxicological endpoints. While effective, these methods may overlook the complex, multifaceted nature of biological interactions, focusing narrowly on specific targets without considering the broader biological context.

Cell Painting (CP) assays mark a significant shift toward a more holistic evaluation of the effects that chemical and genetic perturbations can have on cells. It is based on the assumption that detectable changes in the organization of sub-/cellular structures can serve as indicators for the alterations of normal cell functions. This may be illustrated by the example of facial expression indicating the well-being of a person. Just as a smile or frown can convey underlying emotions, changes in the arrangement of cellular components observed through CP assays can provide valuable insights into the physiological state of the cell in response to various perturbations. CP is an imaging-based high-throughput (HT) phenotypic profiling (HTPP) method that comprehensively captures various cellular phenotypes from sub-cellular compartments and organelles that are visualized by staining with a set of defined fluorescent dyes (Bray et al., 2016; Cimini et al., 2023). Using suitable image analysis tools, hundreds of features representing the specific phenotypic responses of cells to compound treatment can be extracted from the microscopic images. These features translated into a machine-readable format, give rise to so-called *morphological fingerprints*. These fingerprints enable quantitative analysis and “barcode”-like representation of the image data (Nyffeler et al., 2022). The variations in morphological fingerprints are ultimately caused by the mode of action (MoA) of the applied compound treatment. A treatment, thereby, is the application of a *condition* for which one wants to measure the cellular response. These conditions do not necessarily have to correspond to exposures to (pure) compound solutions, but can also represent a metabolically or genetically altered state of the target cells, or treatment with an extract containing a mixture of compounds. Part of the treatment is the incubation time. Using multiple sampling time points and concentrations during incubation allows for capturing dynamic changes in cells, providing valuable insight into their fluctuations over time.

Given the fact that CP experiments can be performed at high throughput, they provide a promising alternative to (binding) assay information, typically used to train machine learning (ML) methods in cheminformatics. Arguably, morphological fingerprints may readily assess what MoA a compound may be acting through in human cells. Such information could, for example, inform the development of anti-tumour compounds (von Coburg and Dunst, 2023) or be used in the assessment of human health or environmental risk of industrial chemicals (Nyffeler et al., 2023). Thus, the introduction of CP opens the route for a paradigm shift towards a more comprehensive assessment of compound effects. However, working with HT cellular readouts also poses significant challenges, which need to be understood and mitigated to make the most use of the data. This review will highlight the advantages of using CP data for predictions, underlining its synergistic relationship with other methodologies and data types. Thereby, it aims to improve mutual understanding of challenges between the wet lab and the dry lab.

## 2 Background on cell painting, molecular representations and artificial intelligence

In the following, first the CP assay will be introduced, as well as the processing of molecular data in a computer-readable format, and finally, how artificial intelligence (AI) currently impacts the field.

### 2.1 The cell painting assay in a nutshell

The concept of *painting* the cells with as many fluorescent morphological markers as possible while maintaining the method’s applicability to large-scale experiments was pioneered at the Broad Institute of Harvard and MIT, with special contributions from the laboratory of A.E. Carpenter (Bray et al., 2016). Since the introduction of the CP concept by Gustafsdottir et al. (Gustafsdottir et al., 2013), the CP assay has been successfully implemented, optimized, and standardized at various sites giving rise to multiple updates of the original method (Bray et al., 2016; Cimini et al., 2023).

#### 2.1.1 Brief description of the general CP pipeline

**FIGURE 1:**
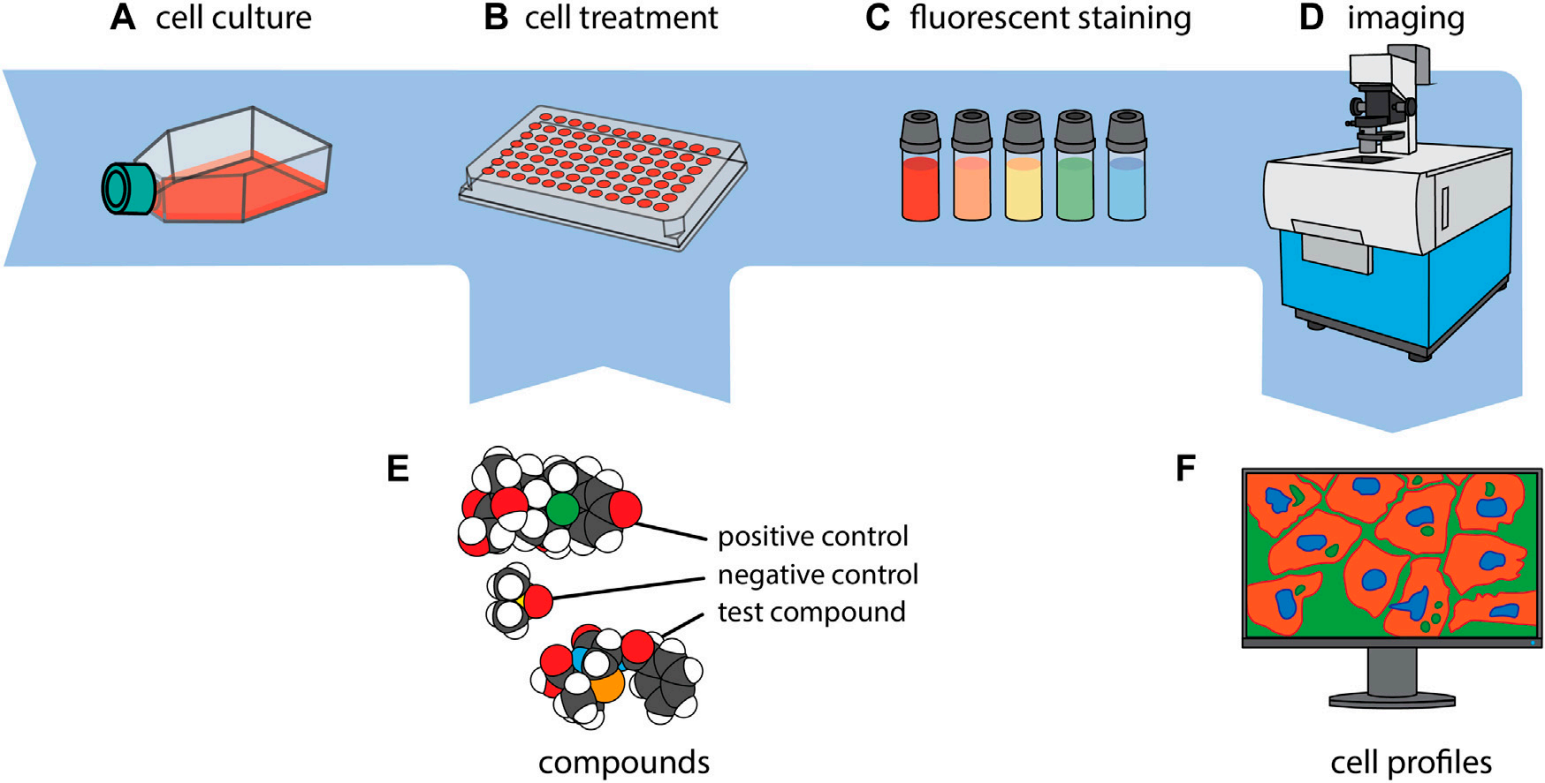
CP in a nutshell. Cells of interest are first cultivated **(A)** and then seeded to, typically, a 384-well plate **(B)**. Every well is exposed to a single compound or genetic perturbation **(E)** and incubated for a period ranging from 24 to 48 h, after which every well is uniformly stained with a collection of fluorescent dyes **(C)**. Subsequently, imaging of the cells is carried out **(D)**, and a phenotypic profile is generated for each experimental condition **(F)**. The images of the molecules in section (E.) were created using CineMol (Meijer et al., 2024).

In the CP assay, cells are typically seeded into 384-well plates, grown for 24 h, and then exposed to different experimental conditions along with suitable positive and negative control reference compounds (Willis et al., 2020; Dahlin et al., 2023) for another 24–48 h. The next step includes multiplexed staining of the cells with six defined fluorescent dyes that label eight distinct sub-cellular compartments and organelles, i.e., DNA, cytoplasmic RNA, nucleoli, actin cytoskeleton, Golgi apparatus, plasma membrane, endoplasmic reticulum and mitochondria (Bray et al., 2016; Cimini et al., 2023). Notably, depending on the imaging system, the limited number of available microscopic channels leads to the merged imaging of at least two dyes (Actin + Golgi), and if four channels are utilized of another two dyes (RNA/ER) in one channel each (Figure 1).

**FIGURE 2:**
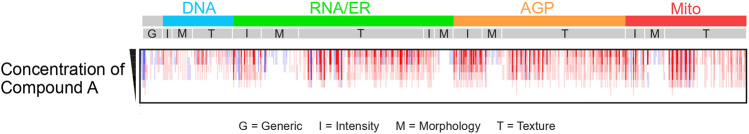
Example of morphological ‘barcode’ representation of CP image data for various concentrations of compound A. Morphological fingerprint includes generic features (G), intensity (I), morphology (M), and texture (T), extracted individually for each channel. Five image channels are depicted: DNA (desoxyribonucleic acid, cyan), RNA (ribonucleic acid) and ER (endoplasmic reticulum, green), AGP (actin, Golgi and plasma membrane, orange), and Mito (mitochondria, red). Features characterize segmented compartments: cells (RNA channel), nuclei (DNA channel), and cytoplasm (excluding nuclei from cells).

Imaging is typically performed using an automated high-content microscope to capture a certain set of image fields. A recent publication by Tromans-Coia et al. (Tromans-Coia et al., 2023) investigated the compatibility of the CP assay with various high-content microscopes and summarized specific recommendations for the implementation of the CP assay to a new laboratory. Each well of the 384-well plate is usually imaged at multiple positions, in horizontal (xy) and vertical (z) dimensions, to collect a sufficiently large number of cells and different sub-cellular regions. Hence, the primary raw output data obtained from CP assays are thousands of multi-dimensional images at gigabyte-scale, with single images typically containing hundreds of cells. During the subsequent image analysis step, these images are computationally processed using automated image analysis tools that are integrated as proprietary software such as Harmony (Revvity Inc.) into the respective high-content microscope or available as open-source software such as CellProfiler (Bray et al., 2016; McQuin et al., 2018; Stirling et al., 2021). Using specific image analysis algorithms, the software corrects image illumination, locates and segments cells employing the Watershed method (Scikit-Image, 2024), and extracts hundreds to thousands of features characterising each single cell. For each individual channel, the extracted features include the size, shape, texture, correlation, and spatial relationships of cells, sub-cellular compartments and organelles (Figure 2).

The nomenclature of CP features typically follows the structure *Compartment_FeatureGroup_Feature_Channel*, although not all features include channel information. Features characterize specific segmented compartments such as cells (identified using the RNA channel), nuclei (defined using the DNA channel), and cytoplasm (identified by removing nuclei from the cells). For each compartment, modules gather unique sets of features, called feature groups, labelled I, G, M, and T in Figure 2. For example, a measure of form factors (metrics characterizing the circular shape of an object, obtained by dividing the area of an object by the square of its perimeter, multiplied by 4 π ) performed on the nuclei will be denoted as follows: *nuclei_areaShape_formfactors*, where *nuclei* is the compartment, *areaShape* represents the feature group or morphological M) module, and formfactors denote the actual computed features.

This computational step produces specific morphological profiles per cell, which can then be aggregated into a profile per experimental condition, essentially serving as a unique morphological fingerprint or signature reflecting the cellular response to the respective chemical or genetic perturbation.

#### 2.1.2 Open-access cell painting image libraries available to the scientific community

The widespread adoption, high throughput and usability of the CP assay led to the deposition of extensive image and profile datasets for community-based analysis. For example, the National Institute of General Medical Sciences (NIGMS) of the National Institutes of Health launched the Cell Image Library (CIL), a database for images and movies of cells from a variety of organisms which contributes to both profiles and images freely accessibility for analysis (Cell Image Library, 2017). In 2017, through the article *“A dataset of images and morphological profiles of 30,000 small-molecule treatments using the Cell Painting assay”*, Bray *et al.* provided the first of such large-scale CP datasets currently available at the CIL (Bray et al., 2017) and the Cell Painting Gallery (Institute B, 2024). Recently, in 2023, the Joint Undertaking for Morphological Profiling Cell Painting (JUMP) Consortium, a collaboration between companies and non-profit institutes, released a dataset of 136,000 chemicals, capturing perturbations for 1.6 billion cells and their single-cell profiles, with an estimated size of 115 terabyte (Chandrasekaran et al., 2023). The Cell Painting Gallery, launched in January 2024, is currently the largest publicly available Cell Painting-specific database with 656 TB in size and includes, among others, those two datasets (Weisbart et al., 2024).

#### 2.1.3 Applications of cell painting in the field of toxicology

As an HTPP method designed for the comprehensive screening of large numbers of compounds, chemicals, or genetic perturbations, CP provides insights into diverse cellular (disease) states by capturing phenotypic responses of cells to perturbations using morphological profiles. The CP assay can be run in single- or multi-concentration approaches to identify active compounds or to derive potency estimates for particular perturbations, highlighting the utility of CP-based phenotypic profiling in regulatory toxicology. For example, CP has already demonstrated its usability for rapid generation of screening-level hazard assessments for thousands of chemicals enabling identification of putative MoAs and grouping of chemicals (Nyffeler et al., 2023). The resulting bio-activity data has been taken up into the U.S. EPA CompTox Chemicals Dashboard. Being an untargeted method, CP enables capturing of a broad array of morphological changes in cells and their organelles without needing specific *a priori* knowledge on potential compound activities for different target organs or tissues. Therefore, CP is an important complementary approach to other profiling methods such as HT transcriptomics (HTTr) analyzing the whole transcriptome. In combination, these two profiling methods can capture changes at different levels of cellular organization, with HTTr focussing on the molecular level (gene expression) and HTPP on the cellular level, enabling concurrent potency estimation and mechanistic prediction of compound activities in a so-called “New Approach Methodology” (NAM)-based chemical hazard evaluation strategy (Thomas et al., 2019; Nyffeler et al., 2022). Moreover, combining HTTr and HTPP data further gives a more holistic view of the cause-and-effect relationship linking a chemical or genetic perturbation with its specific morphological profile. Since CP is conducted *in vitro* using cultured, mostly human cells, it may also serve as an alternative method to reduce animal testing in the future, e.g., as a surrogate pre-screening method to the complex regulatory rodent studies that are still required for evaluating specific target organ toxicity (STOT) upon single or repeated exposure to chemicals for hazard classification and labelling of chemicals (GHS Rev10e, 2023). Importantly, such application scenarios of the CP assay in NAM-based approaches in human regulatory toxicology demand high robustness of the CP assay to provide reproducible data for accurately predicting the hazard of a compound in human cells and require selection of cell types relevant to the biological process under investigation (Schmeisser et al., 2023). For example, a study investigating cardiotoxicity should preferably be conducted using cardiomyocyte cell lines or, at least, cell lines with similar properties (myocytes) rather than other less related cells to ensure translatability of the results.

These datasets are valuable for diverse applications, including studies on 1) the compound MoA as illustrated by Lapins and Spjuth (Lapins and Spjuth, 2019), Schneidewind et al. (Schneidewind et al., 2020), Wong et al. (Wong et al., 2023), Tian et al. (Tian et al., 2023), 2) target identification investigated by Akbarzadeh et al. (Akbarzadeh et al., 2022) and 3) linking cell morphology to disease studied by Cerisier et al. (Cerisier et al., 2023), Lejal et al. (Lejal et al., 2023). Moshkov et al. (Moshkov et al., 2023) demonstrated success in assessing gene function and Seal et al. (Seal et al., 2021; Seal et al., 2022; Seal et al., 2023a) in evaluating environmental toxicants, which will be discussed in more detail throughout this review.

### 2.2 Computational activity or toxic endpoint prediction relies on computer-readable molecular representations

**FIGURE 3:**
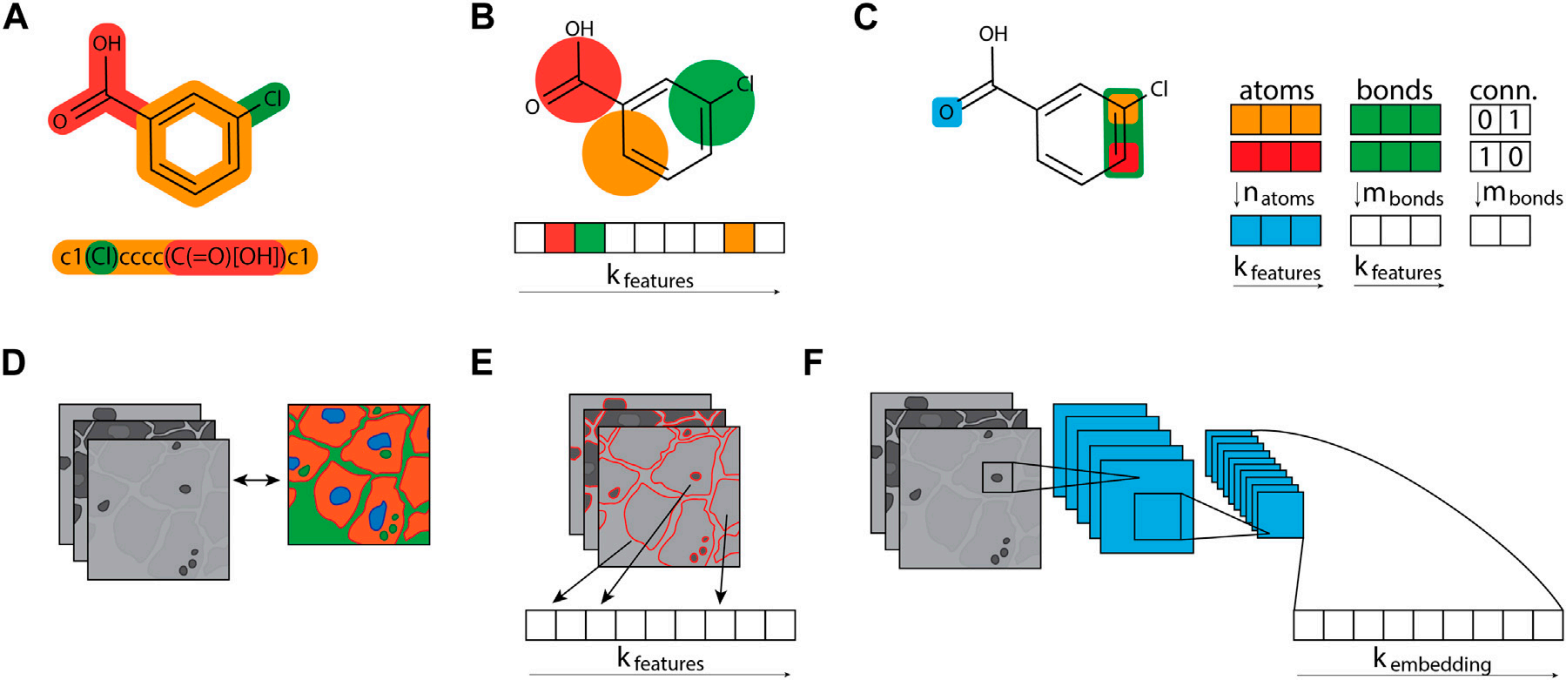
Compound and cell profile featurization. **(A)** Compound structures are typically represented as graphs and stored as compressed text, e.g., as SMILES strings. **(B)** Molecular fingerprint algorithms like a circular Morgan fingerprint can turn the molecular graph structure into an *n*-bit array, converting the molecular graph into a fixed bit-sized array. Fixed arrays are useful for data analysis and machine learning approaches that require tabular data. **(C)** The graph structure can also be leveraged directly for graph neural networks, which keep three distinct tables per compound: a table containing atoms and their features, a table containing bonds and their features, and a table that connects the atoms and bonds. **(D)** Image-based morphological profiles typically consist of multiple grey-scaled images, one image per fluorescent dye used. **(E)** Segmentation tools can process these image profiles and output a fixed-sized array of features. These morphological fingerprints can equally be leveraged by algorithms that process tabular data. **(F)** The morphological images can also be used directly in convolutional neural network architectures, which can *learn* to create a fixed-sized fingerprint, called an embedding.

Computational methods have become an integral part of active compound design, prioritisation and assessment of diverse properties, including toxicity. Computational toxicology concerns predicting the hazards and risks of chemicals for humans, animals or the environment. Commonly used techniques include similarity search, virtual screening, structural alert identification, as well as statistical and machine learning models (Morger et al., 2020; Tang et al., 2023). Especially, quantitative structure-activity and -property relationships (QSAR and QSPR) models play a major key role in predicting molecular properties and toxicity endpoints over the last decade (Muratov et al., 2020; Kusko et al., 2023). The methods rely on the hypothesis that structurally and property-similar molecules exhibit similar behaviour on the target or in the whole cell. To train and apply such methods, the molecules need to be transferred to a computer-readable representation. Thereby, the choice of representation has a high impact on the predictions and diverse types of representations have been introduced over the last decades, as illustrated by Figure 3 (David et al., 2020; Backenköhler et al., 2023).

#### 2.2.1 Representations extracted from the molecular structure

Traditionally, molecules were described by features, mapped into a so-called descriptor vector or fingerprint. Various types of features are available to describe molecules based on their structure - more precisely the contained atoms - and the information that can be calculated from it. Descriptor vectors can contain count-based information (number of rotational bonds, heavy atoms, etc.), physio-chemical properties (solubility, electro-negativity, molecular weight, etc.), adherence to drug-likeness rules (i.e., rule of three or five) (Lipinski et al., 1997), topological index (Wiener, 1947), Randic and Chi connectivity indices (Randic, 1975). Molecular fingerprints are based on the molecular structure as well and characterize, in an often predefined fixed-size vector, the presence or absence of substructures (e.g., MACCS, 166 fragments) or atom environments (e.g., ECFP, often 1024 or 2048 bits) (Rogers and Hahn, 2010). The MACCS (Molecular ACCEss System) key is composed of structural fragments extracted from the two-dimensional representation of 166 predetermined structural keys. Each bit in this vector indicates the presence (Muratov et al., 2020) or absence (0) of a specific substructure in the molecule (Durant et al., 2002). The Extended Connectivity Fingerprint (ECFP), derived from the Morgan algorithm (Morgan, 1965), captures local atom environments. ECFPs can encompass a diverse range of molecular features, which can be further adapted by changing the diameters for the considered circular atom neighbourhood, including stereo-chemical information. ECFPs are versatile and can be utilised for various tasks such as similarity searching, clustering, and more, making them suitable for a wide range of applications.

#### 2.2.2 Learnt representations from strings or graphs

With the advent of deep learning (DL), more advanced descriptors use learnt molecular representation, so-called embeddings. Embeddings are an abstract representation of molecules in a vector space that preserve learnt structural and functional patterns by the model itself. Techniques derived from natural language processing or computer vision, e.g., graph convolutional neural networks (GCNNs) (Kipf and Welling, 2016), are *end-to-end* trainable models that directly learn to extract features from the input representation. End-to-end thereby means that no additional human information or rules are needed and the algorithm learns the patterns *simply* from the data itself. The typical transformer architecture learns from linear - text-like - representations of molecules such as Simplified Molecular Input Line Entry System (SMILES) (SMILES, 1988), InChiIKey (Heller et al., 2013), or Self-Referencing Embedded Strings (SELFIES) (Krenn et al., 2020). Additionally, a graph naturally is an exhaustive and intuitive way of representing molecules: nodes of graphs represent the molecules’ (heavy) atoms while edges represent the covalent bonds between them. Thus, architectures like graph GCNNs can extract local as well as global features from the graph representation of the molecules.

#### 2.2.3 Representations beyond structures

To complement structural information, biological data from *in vitro* or *in vivo* studies, such as binding assays, are helpful to bring additional layers of information, characterising the response of biological systems to perturbation for activity and toxicity prediction. Incorporating experimental data delves into the biological space, enabling the identification of compounds with analogous behaviour in biological systems, regardless of their chemical similarities. Binding assays reveal how well a compound binds to a particular macromolecule, offering insights into its interactions with biomolecules. Given a compound assay matrix, i.e., a number of compounds measured against a number of assays, these assay outcomes can be used as a *biological fingerprint* (Riniker et al., 2014; Helal et al., 2016; Garcia de Lomana et al., 2021). The additional insights gained from *in vitro* methods vary with the analysis conducted. Cell-based assays offer valuable data such as viability, stress response, and immune activation following a treatment, which can serve as supplementary features for drug evaluation. Similarly, micro-physiological systems can provide detailed information at the tissue and organ level, aiding in characterising the organ states after treatment (inflamed, necrotized, etc). Experimental models, like cell culture, organoids, and organ-on-a-chip systems, mimic physiological properties, facilitating compound screening and the exploration of drug-induced toxicity across multiple systems (Liu et al., 2017; Cong et al., 2020; Xu et al., 2020; Trapotsi et al., 2022; Liu et al., 2023).

### 2.3 Recent advances in using artificial intelligence to predict molecular properties

Classical computational methods for molecular activity or toxicity prediction often rely on techniques such as similarity search, clustering, statistical modelling and machine learning. Unsupervised methods, such as clustering, aim to group data points into homogeneous subgroups by optimizing a proximity index using partitioning (Jin et al., 2010), hierarchical, (Zhao et al., 2010) or density-based (Sander et al., 2010) algorithms. This ensures that data points that end up within the same cluster are highly similar to each other and more dissimilar to those in different clusters. Consequently, clustering can discern patterns in a collection without the need for data labels. Nevertheless, coupled with annotation data, this approach is valuable for MoA prediction and target identification (Schneidewind et al., 2020; Akbarzadeh et al., 2022; Pahl et al., 2023). Supervised learning approaches have been pivotal in predicting bio-activity or toxicity by leveraging features extracted from molecular structure and their corresponding measured values or classes. Algorithms like Random Forest (RF), a type of ensemble model for regression or classification, built on decision trees (Kusko et al., 2023), have shown great success in computational MoA prediction (Seal et al., 2021; Seal et al., 2023a; Seal et al., 2023b). Likewise, Support Vector Machines (SVMs) represent a ML technique that can solve both regression and classification tasks. SVMs have been applied to a variety of tasks to identify molecular adverse outcomes (e.g., carcinogenicity, hepatotoxicity) with promising results (Shi et al., 2023). Such models, leverage the features or molecular fingerprints extracted from the molecular structure and their corresponding measured values or classes to make predictions based on the learnt pattern in the data representation.

However, the advent of deep learning (DL) has further propelled *in silico* drug design, allowing for the utilization of other molecular representations such as graphs and strings. Graph convolutional neural networks (GCNNs) (Kipf and Welling, 2016), Long Short-Term Memory networks (LSTMs) (Hochreiter and Schmidhuber, 1997), and transformers (Vaswani et al., 2023) are among the cutting-edge architectures capable of directly learning features from molecular representations. GCNNs operate on graphs using a message-passing mechanism, aggregating neighbouring information to iteratively update each node within the graph. Chemprop (Heid et al., 2024), a prominent example within this domain, has significantly contributed to predicting various chemical properties, including toxic endpoints. This baseline architecture was also used for predicting compound activity from both phenotypic profiles and chemical structures (Moshkov et al., 2023).

Nevertheless, training such DL models necessitates large, well curated, and harmonized annotated datasets. This is challenging in molecular property and toxicity prediction, disciplines in which data is growing but still scarce compared to image or text processing tasks. In addition, standardized protocols are often missing, and inconsistencies in labels among databases exist. DL models need large datasets to effectively learn complex patterns to generalize well to unseen data. To address this limitation, emerging concepts like transfer learning and multi-task learning have shown promise (Allenspach et al., 2024). *Transfer learning*, in particular, facilitates domain adaptation by leveraging knowledge gained from pre-training on extensive unlabelled public datasets, thereby enhancing generalization to novel compounds. The assumption thereby is that the large unlabelled molecular data helps the model to learn the general characteristics of molecules from a large chemical space. During fine-tuning, this knowledge can then be transferred to smaller labelled data sets. Likewise, contrastive learning models assist the learning process by always considering pairs of data points. These models minimize the distance between similar data points while maximizing the separation between dissimilar ones in the embedding space. Contrastive learning has for example, been applied by Sanchez-Fernandez et al. (Sanchez-Fernandez et al., 2023) to directly learn on CP images using a convolutional neural network (CNN). The images are given to the network as tensors of shape, number of images, image dimensions (e.g., height and width) and number of channels. In each layer of the neural network, the image is convoluted (further abstracted) and the information is pooled and thereby hierarchical patterns are learnt in images (Li et al., 2021). CNNs can therefore be used for image segmentation, as alternative to classical segmentation algorithms like the Watershed Algorithm in CellProfiler. Furthermore, *multi-task learning* enables the simultaneous training of multiple tasks using a single model, leveraging shared knowledge among single tasks to improve global performance. If the single tasks of a multi-task model are related to others, the model learns the internal representation of the input data useful for all tasks.

In addition to these advancements, the integration of advanced architectures from other domains, e.g., computer vision, such as variations of autoencoders (Ma Ranzato et al., 2006) and Generative Adversarial Networks (GANs) (Goodfellow et al., 2014), has opened new avenues for molecular discovery. Note that these methods can be applied for *de novo* generation of compound suggestions with desired properties, instead of *only* predicting molecular properties and ranking given compounds. Autoencoders, including Variational Autoencoders (VAEs) (Kingma and Welling, 2022), are a type of neural networks that enable the generation of new data by learning to reconstruct the input from compressed representations. VAEs compress the input images, yielding a condensed representation namely, *the code*, that is given to a decoder that reconstructs the input resulting in a reconstruction of the original image with little information loss. The second type of generative models are GANs which facilitate the creation of plausible data within the task domain. GANs are two-part models, comprising a generator and a discriminator. The generator learns to produce plausible data, while the discriminator learns to differentiate between the generator’s fake and real data. Notably, models like Mol2Image (Yang et al., 2021) and Morphnet (Lee and Welch, 2022) have emerged, harnessing the power of autoencoders and GANs to generate cell images and cell morphology profiles based on chemical compound structures and gene expression data, respectively. The synergy between chemistry, i.e., compound structure, and morphology, i.e., the response of the cell to treatment, holds great potential in optimizing *de novo* molecular design and chemical de-risking.

In the subsequent sections, we will delve into specific studies that leverage the combined knowledge of cell morphology and chemical information to enhance experimental design and *de novo* drug design using AI-driven approaches.

## 3 Exploring integration of molecular fingerprints and cell painting readouts for activity prediction

### 3.1 Datasets and data integration

This section will provide an overview of commonly used CP datasets (Table 1). Highlighting not only chemical perturbation but also different treatment types as gene manipulation and RNAi, and covering the relationship between cellular morphology and molecular perturbation.

**TABLE 1:** Examples of cell painting (CP) datasets used within the literature. For each CP dataset, the number of treatments, the used cell lines, treatment concentrations, and measured time points are listed, as well as data availability.

Name	Treatments	Cell lines	Concentrations ( μ M)	Time points (h)	Open source
CIL [BBBC047]^1^	30,616	U2OS	various	48	Yes
JUMP^2^	116,750	U2OS	0.10, 0.625	48	Yes
Pahl et al.^3^	4,256	U2OS, HeLa	2–10	48	Yes
BBBC021^4^	113	MCF-7	various	24	Yes
(Gupta et al., 2022)^5^	231	U2OS	10	48	Yes
LINCS^6^	1,920	A549, MCF-7, U2OS	10	6, 24, 48	Yes
BBBC025^7^	315	U2OS	10	96	Yes
Akbarzadeh et al.^8^	15,603	U2OS, HeLa	10	24	No
Schneidewind et al.^9^	3,580	U2OS	2, 10	24	No
Recursion^10^	>5,000	HUVEC, HRCE, RPE, U2OS, HEPG2	various	N/A	Yes

Note: The superscript numbers in the Name column are being reused in Table 2. For Pahl et al. (Pahl et al., 2023) dataset, the concentration corresponds to a range varying from 2 to 10 μM. For BBC021 dataset, each of the 113 treatments is screened at eight-point half-log dose. Recursion provides five different datasets of CP images.

#### 3.1.1 A cell image library dataset of 30,616 small molecules

In 2017, Bray *et al.* released a dataset comprising 30,616 small molecules screened on U2OS cells, a human bone osteosarcoma cell line, using the CP assay method. U2OS cells were seeded in 406 multi-well plates (384 wells per plate) and treated with each of the 30,616 compounds in biological quadruplicates or octuplicates. The resulting image dataset is characterised by five fluorescent channels across six fields of view and is publicly accessible on the CIL repository (Cell Image Library, 2017), the Image Data Resource (IDR - Open Microscopy Environment, 2016), the Cell Painting gallery (Institute B, 2024) and the Broad Bioimage Benchmark Collection under BBBC047 accession number (Broad Bioimage Benchmark Collection, 2017). The workflow for illuminating, correcting, and extracting features from these images using CellProfiler can be found on GitHub (Carpenter, 2017). Raw cell-level profiles were measured 48 h post-treatment for the thirty thousand compounds. Various methods, such as averaging features across all cells for each well, can be employed to create population profiles based on the per-cell profile. Both types of profiles are available in the GigaDB repository (Gigadb dataset, 2017). Among the tested compounds, 10,080 originate from the Small Molecule Repository, 2,260 are drugs, natural products, and small-molecule probes from the Broad Institute’s known bio-active compound collection, 269 are confirmed screening hits from the Molecular Libraries Program, and 18,051 are novel compounds derived from diversity-oriented synthesis (Bray et al., 2017). Since its release, up to now, the technique (Bray et al., 2016) and dataset (Bray et al., 2017) have been cited over 800 times (by March 2024), indicating substantial interest in such datasets.

#### 3.1.2 The Joint Undertaking for morphological profiling consortium

The JUMP consortium, comprising ten pharmaceutical companies, six supporting technology companies, and two non-profit organizations, created a CP dataset containing morphological profiles for 116,715 unique, pure compound treatments. Additionally, the dataset includes the phenotypic effects from over-expressing 12,064 genes and 7,795 gene knockouts. Likewise, to the CIL dataset, the 116,715 compounds were tested on U2OS cells. The consortium partners exchanged compounds with each other and ran approximately five replicates of each experiment, performed as one to two replicates at three to five different sites around the world. The compounds consist mainly of synthetic compounds but also contain a smaller subset of natural products. Treatment incubation time was 48 h and treatment concentration was 10μ M except for one partner who tested at 0.625μ M. The dataset includes pre-processed CellProfiler features (Josh Moore, 2023) for all images (McQuin et al., 2018), organized at a per-well level.

#### 3.1.3 In house datasets

As CP has become more accessible over the past years, thanks to the sharing of protocols and open-source image processing software like CellProfiler, various researchers have generated in-house datasets. This enables research groups to screen their own compound libraries under various conditions and cell types, facilitating the study of molecule-specific modes of action. This approach was exemplified by Pahl et al. (Pahl et al., 2023). They followed the original method described by Bray et al. (Bray et al., 2016) and introduced a different cell line, the HeLa cells (cervix carcinoma), infected with papillomavirus. Various concentrations of 4,256 compounds were tested on both U2OS and HeLa cells, expanding the scope of their investigations for consensus profile analysis and bio-activity annotations. Similarly, Gupta et al. (Gupta et al., 2022) followed the protocols established by Bray et al. to produce five-channel CP images for MoA prediction. This dataset was created for model development for Tian et al. (Tian et al., 2023). One notable advantage of generating in-house images lies in the control over parameters, including image resolution and plate layout design. In this context, they employed a tool called PLAID (Plate Layouts using Artificial Intelligence Design) (Francisco Rodríguez et al., 2023) to achieve an optimized plate layout that minimizes plate effects. Other datasets from the Broad Institute, using different cell lines and treatment as gene expression can be accessed at the cell painting gallery (Institute B, 2024). Note, this is just an extract of in house data sets included in the studies discussed in this review, with no claim to completeness.

#### 3.1.4 Omics data integration

Combining the read-outs from different types of treatments has the ability to give a holistic insight into the cause-and-effect relationship between different treatments and the cells’ change in morphology. Apart from compound treatments, consideration can be given to the relationship between cell morphology and a given treatment at the genetic, transcriptomic, proteomic, and/or metabolic level, as measured by the respective *omics* technique. For example, genomic data, such as DNA sequencing or gene expression profiles, can help link phenotypic changes observed in the assays to genetic alterations. Performing CP assay on different cell models with knock-down and knock-out genes could give insight into identifying drivers of diseases, and cellular responses to drugs. Chandrasekaran et al. (Chandrasekaran et al., 2022) assessed genetic perturbations in addition to some of the compound treatments by including 176 gene over-expression and 160 gene knock-down treatments. They combined these morphological profile data with datasets from Recursion (Sypetkowski et al., 2023) which is a comprehensive, and largely proprietary dataset describing compound and genetic perturbation treatments. In the same manner, integrating transcriptomic data can help correlate changes in gene expression with phenotypic alterations, and identify transcriptomic profiles within target cells that are associated with specific MoAs of the compounds to which they are exposed (Carraro et al., 2022). Protein levels can be measured alongside CP, providing detailed insights into how genetic modifications, compound treatments, or disease states influence cell morphology. This is especially valuable for identifying compounds that bind to key regulatory proteins (Ma Y. et al., 2006). Next to the proprietary Recursion dataset, multiple open-source datasets containing CP data from different treatment types have been created. Primary examples are the LINCS dataset, created by Keenan et al. (Keenan et al., 2018) and Natoli et al. (Natoli et al., 2021), and the recently created JUMP dataset by Chandrasekaran et al. (2023).

### 3.2 Approaches for molecular activity and toxicity prediction

**FIGURE 4:**
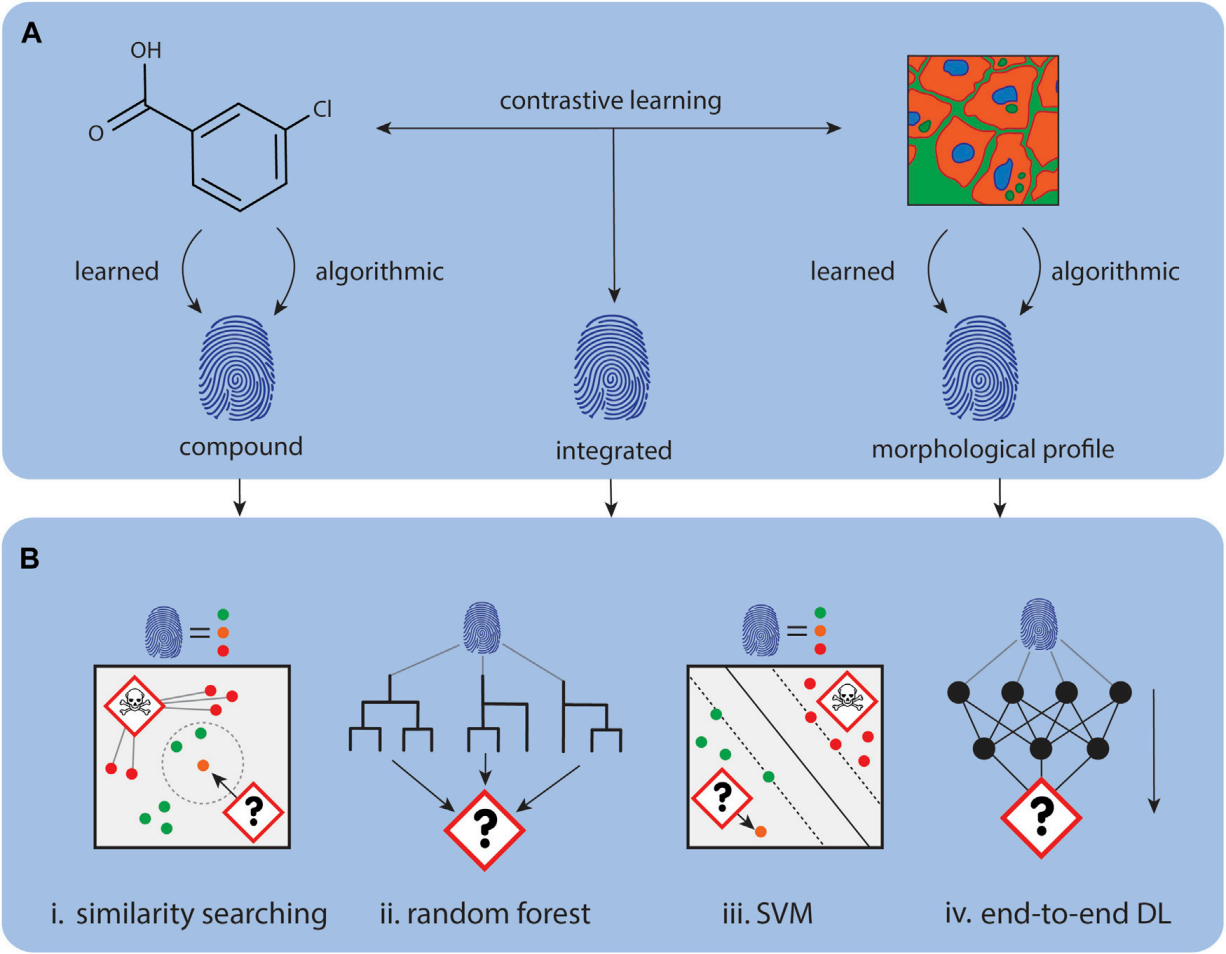
Using CP data for mechanism of action prediction. **(A)**. Representations for compounds and CP images can be either calculated, for example, with CellProfiler, or learnt with deep learning approaches. **(B)**. Data representations can be concatenated or used independently for downstream tasks. **(i)** The MoA of new representations can be determined through comparison with similar annotated representations. **(ii)** Tree models like Random Forest’s can be used to predict MoA for new representations. **(iii)** Other classical machine learning algorithms like SVMs can also learn to predict MoA for new representations. **(iv)** Instead of using the representations for downstream tasks, a predictor module can be directly attached to model learning the representations to create an end-to-end predictor.

In the following, the state-of-the-art methods including morphological data for activity and molecular property prediction, including toxicity, are discussed. The general workflows on how the data can be integrated into the individual models is exemplified in Figure 4.

#### 3.2.1 Similarity search and hierarchical clustering for annotation and MoA prediction

In this section, we explore how *bio-similarity* of morphological fingerprints/profiles serves as a metric for clustering and similarity search to annotate and identify the MoA of novel compounds. Bio-similarity of morphological fingerprints/profiles is defined as one minus the correlation distance between two profiles (Eq. 1).

$$\begin{array}{c} dist_{\text{corr}}\left(x,y\right)=1-\frac{x^{⊤}y}{\left\|x\|\cdot \|y\right\|}\\ \text{BioSim}=1-dist_{\text{corr}} \end{array}$$ (1)

Where $x^{⊤}y$ represents the dot product of two morphological fingerprints x and y ; ‖x‖ and ‖y‖ denote the Euclidean norm (magnitude) of profile x and y , respectively.

Schneidewind et al. (Schneidewind et al., 2020) used morphological fingerprint similarity to deduce the mode of action of small molecules. A morphological fingerprint from a compound with a known mode of action is taken as a reference, and the annotation is then extrapolated to closest match(es) in terms of bio-similarity. The study is performed with deferoxamine (DFO) as the reference, an iron-chelating agent known to disrupt cell cycles. The authors seek matches within 3.580 morphological fingerprints of annotated molecules. Consequently, molecules with a bio-similarity score to DFO exceeding 80% are deemed to share the same mode of action as DFO. The hypothesis is validated through case studies; e.g., ciclopirox and phenanthroline, two known metal ion chelators, were found to have a high bio-similarity of over 93% to DFO. Also unexpected molecules displayed high bio-similarity to DFO. An example is PAC-1, a procaspase activator, with an 89% bio-similarity to DFO, despite targeting a different protein. Further investigation revealed that PAC-1 activates procaspase3 by chelating zinc ions. Similarly, 20 other molecules exhibit high bio-similarity (79%–92%) to DFO but were annotated as nucleoside analogues, CDKs, topoisomerases, DNA intercalating agents, and others. Iron serves as a crucial factor in cellular life (Yu and Richardson, 2007); enzymes involved in DNA repair and synthesis require iron as cofactors (Puig et al., 2017). Additionally, iron can inhibit cell proliferation and induce cell-cycle arrest in the G1/S-phase (Siriwardana and Seligman, 2013), modulating the expression of various cyclins and cyclin-dependent kinases, thereby leading to cell-cycle arrest. Thus, the similarity among these diverse compounds does not arise from the modulation of a single target, but rather from a shared mode of action, namely, inducing cell-cycle arrest. Consequently, Schneidewind *et al.* demonstrated that bio-similar but structurally distinct compounds with varying annotated cellular targets can cause shared physiological responses.

Likewise, Akbarzadeh et al. (Akbarzadeh et al., 2022) employed morphological fingerprints to identify compounds targeting tubulins, emphasizing the importance of characterizing off-target binding. *In vitro* tubulin binding assays may not accurately reflect the cellular state, leading to insufficient characterization of tubulin binding mode due to scarce high-quality phenotypic assays. Identifying common morphological patterns, induced by small molecules binding tubulin, helps in predicting and experimentally validating microtubule-binding activity. Utilizing nocodazole’s morphological profile as a query—a known tubulin-targeting agent—known drugs, e.g., vincristine and vinblastine, with the same MoA were successfully identified. Microtubule targeting is crucial in cancer therapy, but the multifaceted modulation of tubulin by small molecules complicates compound development. Akbarzadeh *et al.* demonstrated that bio-similarity search using nocodazole’s CP profiles helps in predicting microtubule modulation for unexplored compounds and several reference compounds initially unrelated to tubulin. Experimental evidence, including live cell imaging, supported this conclusion, highlighting the need for early investigation of tubulin as an off-target in compound development.

Pahl et al. (Pahl et al., 2023) analyse sub-profiles of morphological fingerprints to annotate novel molecules. A sub-profile is a consensus fingerprint that contains only a subset of features characterizing a set of bio-similar molecules. Such sub-profiles are generated by first conserving features with the same sign (positive or negative) and then aggregating them into one fingerprint by taking their median. The set of bio-similar molecules refers to compounds having the same known MoA, e.g., tubulin inhibitors. Therefore, Pahl et al. generated consensus fingerprints, i.e., sub-profiles, defining 10 MoA groups. As the sub-profiles use a subset of features, these features help in allocating molecules to the same MoA group. For instance, in Schneidewind et al. (2020)’s study, the common MoA identified using DFO's morphological fingerprint as a query is cell cycle impairment. This MoA group also contains molecules that inhibit CDKs, affect topoisomerases, and are nucleoside-type. A hierarchical clustering based on the selected features reveals a distinct separation among compounds sharing the same MoA, grouping them into (sub-)families. Thus, Pahl *et al.* were able to assign unknown molecules to known MoA clusters using their sub-profile similarity and experimentally confirmed their findings by using three uncharacterized compounds and mapping them to a cluster of molecules that impact protein synthesis similarly to DFO.

*Summary:* Binding assays address the modulation of proteins by small molecules, however, they do not reflect their influence on whole cell processes. Schneidewind et al. (Schneidewind et al., 2020) illustrate that compounds with diverse annotations form morphological clusters which are based on the same mode of action rather than on the same target. Furthermore, bio-similarity between compounds helps to unveil the MoA for unexplored compounds and to identify un-expected off-target activities for known compounds, as demonstrated with Akbarzadeh et al. (Akbarzadeh et al., 2022) and Pahl et al. (Pahl et al., 2023).

#### 3.2.2 Random forest for the mean of toxicology and MoA prediction

A RF model aims to follow a path of criteria along decision trees - learnt from the input features (e.g., gene expression data, morphological or molecular fingerprints) of a set of training compounds - to predict a specific outcome, e.g., cytotoxicity. The final decision is made at the leaf node level, e.g., for cell viability, toxic vs non-toxic. Thereby, decision trees identify effective feature splits to segment the data. By aggregating multiple decision trees, the random forest algorithm improves predictive power. The predicted class for a data point is determined by the most frequently occurring categorical variable among the decision trees, addressing the initial question, i.e., about the molecule’s cytotoxicity.

Seal et al. (Seal et al., 2021) compared the performance of RF models, incorporating morphological fingerprints from Bray et al. (Bray et al., 2017) (see Table 1, BBBC047) and structural fingerprints to predict proliferation-related assay outcomes obtained from the MoleculeNet ToxCast benchmark (Wu et al., 2017). The study investigated whether single or combined modalities, i.e., molecular, morphological, or combined fingerprints, better predict cell viability. A RF model was built and optimized separately on each type of fingerprint through nested k-fold cross-validation and leave-one-cluster-out (LOCO) split (see methods of Seal et al. (Seal et al., 2021)). Permutation importance analysis was employed to identify the contributing morphological fingerprint features for predicting proliferation assay outcomes. Seal *et al.* explored two different structural fingerprints (Morgan and Extended reduced Graph) with mediocre prediction performance, e.g., average balanced accuracy for LOCO split is 0.56 ± 0.13 and 0.54 ± 0.09, respectively, for the *BSK_3C_Proliferation_down* endpoint (refer to Table 2 in Seal et al. (Seal et al., 2021)). Cell painting data alone proved more effective in predicting 9 out of 12 studied cases, i.e., assay outcomes related to cell viability or proliferation, with a mean balanced accuracy of 0.66 ± 0.1. However, combining morphological and Morgan fingerprints yielded the best result in 10 out of the 12 studied cases with a mean balanced accuracy of 0.77 ± 0.12. Additionally, morphological feature contributions were extracted for different endpoints to enhance the interpretability of the results. Compared to structural fingerprints, morphological fingerprints can be linked to MoAs or to identify novel markers for, e.g., cell viability/toxicity. Specifically, after cell death, remaining cells had reduced contact with neighbours, leading to contracted edge pixels touching another cell, thus, identifying cell neighbour features as crucial indicators of cell death/viability markers in the assays.

**TABLE 2:** Summarizes the state-of-the-art discussed works together with the employed methods, the cell painting (CP) data used, and the prediction task they were applied to. The superscript numbers in the CP Data column refer to the data set summaries in Table 1. Method abbreviations: Random Forest (RF), Neural Network (NN), Feed-forward NN (FNN), Convolutional NN (CNN), conditional Generative AdversarialNetwork (cGAN).

Authors	Method	CP Data	Application
Schneidewind et al. (2020)	Similarity search	in house^9^	MoA annotation
Akbarzadeh et al. (2022)	Similarity search	in house^8^	Target annotation
Pahl et al. (2023)	Similarity search and clustering	in house^3^	Target Annotation
Seal et al. (2021)	RF	BBBC047^1^	Assay activity
Seal et al. (2022)	RF	BBBC047^1^	Mitochondrial toxicity
Seal et al. (2023a)	RF	BBBC047^1^	Cardiotoxicity
Moshkov et al. (2023)	Chemprop	BBBC047^1^	Assay activity
Simm et al. (2018)	RF, *k*-NN, Macau, FNN	in house	Assay activity
Hofmarcher et al. (2019)	FNN, CNN	BBBC047^1^	Assay activity
Wong et al. (2023)	CNN	in-house^5^	Assay activity
Tian et al. (2023)	FNN, CNN, classical ML	in-house^5^	Assay activity
Nguyen et al. (2023)	Contrastive learning	JUMP^2^	Molecular property prediction
Sanchez-Fernandez et al. (2023)	Contrastive learning	BBBC047^1^	Assay activity, cross-modal retrieval
Gabriel et al. (2023)	Contrastive learning	JUMP^2^	Cross-modal retrieval
Zapata et al. (2023)	cGAN	BBBC047^1^	Molecular generation
Palma et al. (2023)	Autoencoder	BBBC021^4^	CP image generation

Drug-inducing mitochondrial toxicity frequently contributes to late-stage withdrawals of marketed therapeutics. Therefore Seal et al. (Seal et al., 2022) explored predicting mitochondrial toxicity by integrating gene expression profiles (Links: L1000 dataset (Keenan et al., 2018)), morphological (from the CIL dataset, (Bray et al., 2017), BBBC047, Table 1) and Morgan fingerprints (derived from molecules using RDKit (Team and Landrum, 2024)). The molecules within the dataset are labelled as potential mitochondrial membrane disruptors using the Tox21 dataset endpoint with assay ID *AID 720637*. The overlap between the gene expression and the morphological datasets with the mitochondrial toxicity annotation results in a dataset of 404 unique molecules. In addition to this, an external test set is used from Hemmerich et al. (Hemmerich et al., 2020) plus 8 molecules from Mitotox (Lin et al., 2021). Five RF models were built to predict mito-toxicity; one for each input type (gene expression, morphological and structural fingerprint), one *early fusion* model (the features are concatenated into one vector and given as input to the model), and one *late fusion* model (the probabilities of the predictions of the three single RF models are averaged). A multitude of disrupting mechanisms can result in mitochondrial toxicity, by identifying links within the different input types covering different domains, one can gain more interpretable insight into the cause leading to mitochondrial toxicity. Seal et al. first evaluated the informative power of CP features and structural fingerprint through morphological (Pearson correlation) and fingerprint (Tanimoto coefficient) similarity analysis. Mitochondrial disruptors exhibit greater morphological than structural similarities, indicating that morphological fingerprints are more effective in discriminating between both compound classes. After conducting principal component analysis using morphological fingerprint (110 features post-reduction), clusters representing various causes of mitochondrial toxicity were identified. Some clusters gathered known microtube destabilisers (e.g., benzimidazoles) with structurally different molecules (e.g., rotenone). Additionally, certain gene features are associated with mitochondrial toxicity (e.g., endoplasmic reticulum stress, T cell apoptotic process) correlated with morphological features (e.g., *Cytoplasm_AreaShape_FormFactors*, Figure 2), suggesting that morphological readouts also reflect mitochondrial toxicity pathways (Szegezdi et al., 2006). Based on this insight, RF models were trained similarly as described in their earlier study (Seal et al., 2021). Despite decent performance of both morphological and gene expression data (balanced accuracy (BA) 0.6), RF models built on Morgan fingerprints alone showed lower performance, with a drop in BA from 0.69 to 0.58 and from 0.57 to 0.2 between training and external test sets. The *late-stage* fusion RF model seemed to be the most promising, correctly predicting more mitochondrial toxic compounds with BAs on the training and external sets of 0.7 and 0.69, and with a sensitivity of 0.62 and 0.78 on those sets, respectively.

Likewise, Moshkov et al. (Moshkov et al., 2023) combined the three same modalities (gene expression from L1000, CP data from Bray et al., structural information derived from Chemprop). Although the architecture of the model used in Moshkov *et al.* is different, the authors came to a similar conclusion. Morphological fingerprint and gene expression bring complementary information, and phenotypic profiles improve prediction performance compared to models built on structural fingerprint information only.

In 2023, Seal et al. (Seal et al., 2023a) conducted another study exploring the potential of chemical and biological data in predicting drug-induced cardiotoxicity. Here, the investigated feature space was further extended, including phenotypic information (such as CP, gene expression, and gene ontology data), binding assay-related data (including how a drug interacts with various biological targets (inhibition/antagonism information)), pharmacokinetic data (as the total and maximum unbound and total concentrations (Cmax) in plasma), structural descriptors (Morgan fingerprint, physiochemical descriptors), and target-MoA annotation. A total of 11 RF classifiers were trained using either individual features or combinations to analyze the contribution of the individual feature spaces. The six best-performing RF models (including structural and physicochemical descriptors, MoA, Cmax, MoA-Cmax, and binding assay-related data), were selected to create an ensemble model, the final prediction is determined by averaging probabilities across the six models, known as soft voting. Additionally, a second ensemble model that considers all features individually was built with both models aiming to predict cardiotoxicity. The ensemble model, using the six best-performing feature sets, achieved results equivalent to the model built on the structural descriptors alone, with an Area Under Curve (AUC) of 0.83. The various analyses conducted revealed key features associated with drug-induced cardiotoxicity. For instance, analysis using morphological data revealed that features related to the endoplasmic reticulum (ER) and RNA texture, measured in both the cytoplasm and the nucleus played a significant role in distinguishing between toxicity classes. These observations align with (Belmadani and Matrougui, 2019) indicating that morphological texture features may signify disruptions in ER function leading to ER stress, a condition associated with various cardiovascular diseases.

In Lapins and Spjuth (Lapins and Spjuth, 2019), the study similarly integrates the same three data domains, as mentioned before, to conclude on a synergistic predictive power. However, the study also emphasises the importance feature pre-processing can have for model accuracy and interpretability.

*Summary:* These papers highlight the advantageous synergistic effect of integrating information from distinct domains for both toxicity-related tasks and MoA/target annotation. Seal et al. (Seal et al., 2021; Seal et al., 2022; Seal et al., 2023a) highlight the significance of CP data for MoA annotation by establishing connections between several data domains and interpreting the outcomes derived from RF models, thus, providing mechanistic insights into toxicology prediction.

#### 3.2.3 Learning directly on cell painting images

CP images can be represented as three-dimensional tensors, with the dimension length of image x width of image x number of channels. In this data structure, one unit is defined by one pixel. The spatial arrangement of the pixels in this data structure has a meaning. When converted to features in a morphological fingerprint, this spatial information is lost. But these one-dimensional representations can be processed by classical machine learning algorithms (e.g., RF and SVM) or feed-forward neural networks (FNNs) (Svozil et al., 1997). However, for the model to learn on the spatial arrangement of the features (i.e., pixels) as well, specialized algorithms like CNNs (Li et al., 2021) are required.

Simm et al. (Simm et al., 2018) collected bio-activity data from an image-based phenotypic screen on 524,371 proprietary compounds, specifically a three-channel microscopy-based screen for glucocorticoid receptor translocation, and collected response classes for these compounds based on their activity on diverse protein targets. The authors retained classes when there were at least 25 compounds with a positive response and 25 compounds with a negative response. This resulted in 545 target classes. CellProfiler was used to extract features from the phenotypic screen. The authors used various machine-learning models, including RF, *k*-Nearest Neighbours (kNN), Bayesian matrix factorization (Macau) and FNNs, to predict the aforementioned bio-activity classes of these compounds. The authors deemed a prediction task for an assay to be successful if the model was able to predict the correct bio-activity of compounds with an AUC-ROC of at least 0.9. The FNN performed best with 8% successful assay prediction tasks (43 out of 545), followed by Macau with 5.8%. RF and kNN resulted in 2.3% and 0% success rates, respectively. To test if the best-performing models were able to predict the bio-activity for a new target, Simm et al. (Simm et al., 2018) performed two *in vitro* tests: one against an undisclosed kinase, and another against an undisclosed non-kinase enzyme. For these *in vitro* tests, the authors used the Macau model to predict compound bio-activity. In the first test against the kinase, the authors used the Macau model to predict the bio-activity of 60,000 compounds from the original phenotypic screen that had no bio-activity data on the respective kinase. 342 highest ranking hits were selected for wet-lab testing, which resulted in 124 hits (i.e., XC_50_ <1μ M). In the second test against the non-enzyme kinase, the Macau model was used again to predict the bio-activity of all 524,371 compounds from the original phenotypic screen. After filtering-out the 141 high-ranking hits compounds, 36 hits were selected for wet-lab testing.

Hofmarcher et al. (Hofmarcher et al., 2019) showed that using a CNN trained directly on the images, provided a substantial increase in performance over training on morphological fingerprints. In this study, the authors annotated compound-image profile data pairs from the Cell Painting dataset by Bray et al. (Bray et al., 2017) with activity labels mined from ChEMBL. Consecutively, they used various CNN-based architectures to predict these activity labels solely based on the images. The findings indicate that CNNs significantly outperform FNNs operating on precomputed morphological fingerprints. The best-performing CNN was able to predict the outcome of 32% of 209 biological assays with an AUC higher than 0.9, compared to the FNN which achieved tis in 26% of the cases.

To test the performance of CNNs on image-based morphological profiles, Wong et al. (Wong et al., 2023) created MOAProfiler, a modified pre-existing CNN-based EfficientNet (Tan and Le, 2019) image classifier model that predicts MoAs for five-channel images from CP assays. For the training data, the authors collected compound-image profile data from the JUMP pilot dataset (Chandrasekaran et al., 2023) and LINCS (Natoli et al., 2021), and labelled them with MoA classes when the annotation was known. Only the compounds with at least one label were retained for training. This resulted in 266 compounds with 176 unique MoA annotations from the JUMP pilot dataset for training and a subset of 56 MoA annotations for testing. In addition, 1287 compounds with 424 unique MoA annotations from the LINCS dataset for training and a subset of 215 MoA annotations for testing. Separate models were trained for both datasets. The authors achieved an Area Under Precision-Recall Curve (AUPRC) of 0.46 with MOAProfiler on the JUMP pilot dataset hold-out MoAs and an AUPRC of 0.34 on the LINCS dataset hold-out MoAs. Additionally, the authors extracted embedding layers from both of the trained models and aggregated these. These embeddings represent learnt representations of the CP and compound input data. Wong et al. (Wong et al., 2023) compared the extracted image profile embeddings from MOAProfiler with extracted image profile embeddings from DeepProfiler (Bornholdt et al., 2022) and with CellProfiler fingerprints. The embeddings and fingerprints were compared by calculating average Pearson correlation coefficients between embeddings for the same MoA, performing *k*-Nearest Neighbours classification for well-level MoA, and a custom class latent assignment metric. This metric assigns MoA to a test set sample embedding by calculating which median-aggregated MoA-level embedding is closest to it in embedding space. The median-aggregated MoA level embedding is obtained by retrieving all training embeddings related to a specific MoA and calculating the median for every feature. For the models on both datasets, MOAProfiler outperformed the DeepProfiler embedding and the CellProfiler fingerprint on all accounts. This indicates that embeddings generated by MOAProfiler are more distinguishable per MoA.

Tian et al. (Tian et al., 2023) used a similar approach as MOAProfiler, a modified EfficientNet model for directly learning on the CP images. Instead of predicting MoA on the images alone, the authors created a model architecture that also incorporated the molecular features of the compound used for the treatment to create each CP profile, in order to complement the image data. The modified EfficientNet model was trained on 231 annotated image-based morphological profiles, while the compound classifier was trained on 5500 annotated compounds. The image data accompanying this paper is described by Gupta et al. (Gupta et al., 2022). The authors tested several different models for classifying the compounds, including classical machine learning algorithms trained on Morgan fingerprints, an FNN trained on Morgan fingerprints, and a GNN trained on the molecular graphs. When trained on only the molecular data, the best-performing model, the FNN, achieved a macro-averaged F1 score (i.e., unweighted mean of all per-class F1 scores) of 0.58. Training on only the cell morphology image data with the modified EfficientNet model resulted in a higher macro-averaged F1 score of 0.81. The global model, which was trained on both types of data, achieved the highest macro-averaged F1 score of 0.92, indicating a clear synergistic effect from combining these data sources.

The previous examples focused on predicting MoA from compounds and image profiles directly. These models require MoA annotations for every training sample. This limitation can be overcome by using unlabelled compound-image profile data pairs for contrastive learning. Nguyen et al. (Nguyen et al., 2023) created a multi-modal contrastive learning framework called Molecule-Morphology Contrastive Pretraining (MoCoP), that integrates molecular graph data and cellular morphology. The authors featurized CP image data from the JUMP dataset with CellProfiler (Chandrasekaran et al., 2023) and used that data to contrastively pre-train a GNN for molecular property prediction. To evaluate the pre-training, Nguyen et al. (Nguyen et al., 2023) measured the accuracy of molecule and image profile retrieval tasks using the JUMP dataset. The retrieval performance was quantified by reporting the average top-*k* accuracy for retrieving a molecule given its morphology and *vice versa*. MoCoP is able to retrieve the correct (i.e., top-1) sample among 1000 randomly picked samples in about 5% of the cases when pre-training is performed on 101,000 compounds, and in about 20% of the cases when the correct sample is among 100 randomly picked samples. Subsequently, the pre-trained GNN was evaluated through transfer learning on 1310 binary downstream tasks (number of classes), for approximately 450 thousand compounds, described in the ChEMBL20 dataset. ((Gaulton et al., 2012)) The pre-trained GNN showed an average improvement of 2.6% and 6.3% in AUPRC for full and low data regimes (i.e., low data regimes use a subset of all the training data for training), compared to the baseline model, also a GNN, that was trained on the downstream tasks only. The results show that contrastive pre-training improves the performance of molecular property predictors, especially when more compounds are included.

The Contrastive Learning and leave-One-Out-boost for Molecule Encoders (CLOOME) model, created by Sanchez-Fernandez et al. (Sanchez-Fernandez et al., 2023) follows a similar strategy. The authors used compound-image profile pairs from the Cell Painting dataset (Bray et al., 2017) to create a shared embedding. However, instead of using CellProfiler features to represent the cell profile images, Sanchez-Fernandez et al. (Sanchez-Fernandez et al., 2023) operate on images directly during model training. To evaluate the information content of the embeddings, the authors tried to retrieve images with compounds and *vice versa*, just like Nguyen et al. (Nguyen et al., 2023). On a hold-out dataset of 2115 compound-image pairs, CLOOME was able to correctly retrieve the compound from the image in 3.8% of the cases, and retrieve the image from the compound in 3.2% of the cases. The transferability of these learnt compound-image embeddings were highlighted by using them as training data for 209 downstream bio-activity prediction tasks. These are the same evaluation tasks as performed by Hofmarcher et al. (Hofmarcher et al., 2019). CLOOME was able to predict the outcome of 27.3% of the 209 biological assays with an AUC higher than 0.9, which is a little bit lower than the 32% by the CNN-based models created by (Szegezdi et al., 2006). However, it should be noted that CLOOME was trained without the need of MoA labels, which allows for more (unlabelled) data to be used for training.

Gabriel et al. (Gabriel et al., 2023) introduced two novel loss functions for improving contrastive learning on compound-image pairs: Extra Modality Multiview (EMM) and Intra Modality Multiview (IMM). Loss functions are used in deep learning to steer the learning process by minimizing the difference between the predicted output of a model and the actual outcome (or label). EMM focuses on the relationships between a compound and images, while IMM considers the relationships between images. The authors used their newly devised loss functions to train a Contrastive Language-Image Pre-training (CLIP) model architecture ((Radford et al., 2021)), using the JUMP dataset ((Chandrasekaran et al., 2023)) as training data. The CLIP model with EMM loss function was able to correctly use an image to retrieve its paired compound from a pool of 100 compounds in 8.5%, and for IMM in 9.6% of the cases, and in 10.3% using EMM and in 7.7% using IMM for retrieving the correct image from a compound. This is a modest improvement over using the CLIP model without the EMM and IMM loss functions, which was only able to retrieve the correct compound for an image in 7.7% of the cases, and the correct image for a compound in 6.6% of the cases. Moreover, the authors show that the EMM and IMM loss functions can be used to mitigate batch effects in the data. Batch effects are variabilities in the data due to differences in experimental conditions rather than biological factors. Presumably, mitigating batch effects will increase the quality of learnt embeddings, improving downstream prediction tasks as well. Such batch effects might arise when CP data is collected from several different sources. The authors quantified batch effects by calculating the performance of k-Nearest Neighbours and logistic regression classifiers on several train and test data splits stratified by source (e.g., CP assay location), batch (e.g., time point or microscope used), and plate. For these classification tasks, Gabriel et al. (Gabriel et al., 2023) trained classification methods to predict a compound’s target gene. Batch effect mitigation was measured by evaluating the ratio between the performance of a model trained on one of the stratified train and test data splits and the accuracy of a model trained on a random train and test data split. The improvement (ratio of better-performing models) using EMM and IMM was, respectively, 0.83 and 0.93, while the ratio for CLIP without using the novel loss functions was 0.72—a clear improvement.

Summary: CNN-based models, leveraging CP image information directly, show improved performance in predicting biological assay outcome over FNNs and classical machine learning methods leveraging morphological fingerprints. Furthermore, learnt multi-modal representations through contrastive learning on images and compounds can be used effectively for multiple downstream tasks like similarity searching, cross-modal retrieval, and classification.

#### 3.2.4 *De novo* design: Generative modelling for tailored molecules and phenotypes

Zapata et al. (Zapata et al., 2023) and Palma et al. (Palma et al., 2023) both observed that the application of CP data together with small molecules primarily revolved around clustering and classification tasks on either extracted features or images. While these methods are useful for the prediction of molecular properties for given molecules, the next step is to directly predict new molecules with the desired properties. Consequently, both authors introduce proof-of-concept studies integrating CP data into generative AI approaches, proposing a novel direction for the use of morphological data.

On the one hand, Zapata *et al.* leverage information from CP profiles to guide *de novo* design. Their model consists of two parts: first, a VAE that takes SELFIES as input and creates a molecular embedding of the SELFIE. This model generates molecular embeddings (encoder) and is trained to reconstruct discrete representations of compounds (decoder). The training is conducted on ChEMBL22 to cover a broad chemical space. The second part of the model is a conditional Generative Adversarial Network (cGAN). This cGAN is trained on the BBBC047 dataset and utilizes the well-level profiles, totaling 126,779 profile (Table 1). The cGAN takes a vector of noise and a morphological profile as input to generate an embedding (generator). The discriminator calculates the probability of the embedding coming from a profile from a real molecule or a generated profile, while the condition network evaluates the probability of the embedding matching the input morphological images. The previously trained decoder unit part of the VAE takes the generated molecular embedding to translate them into SELFIES. Zapata *et al.*‘s model generated 30,000 molecules conditioned on an equal number of profiles, of which 15,000 were deemed valid and unique. The generated molecules exhibit low similarity and high scaffold diversity compared to the training set, highlighting the novelty of the compounds. Drug likeness was evaluated by computing Lipinski’s rule of five and QED scores, indicating that, on average, the molecules possess drug-like properties. The Retrosynthetic Accessibility score, a metric which estimates synthetic feasibility (Thakkar et al., 2021), exceeded 0.5 for over 50% of the generated compounds, with less than 25% containing toxicophores. Ideally, such a model would help to design molecules with specific bio-activity toward a protein or help in optimizing molecular properties.

Palma *et al.* learnt from small molecules and morphological features to infer morphological responses to treatments. The Image Perturbation Autoencoder (IMPA) model employs a style-transfer approach, which involves altering the style (perturbation) of an image while preserving its original content (cell representation). The model is trained to distinguish between untreated cells and cells treated with a perturbation (genetic or molecular). Training is conducted on a subset of the BBBC021 dataset (five molecules), using breast cancer cell lines (MCF-7); images are cropped for every single cell, resulting in 97.000 data points. Likewise, the BBBC025 dataset contains 350 gene perturbations and the dataset is used for training. The style encoder links image features to a perturbation-specific style and can be used to inspect cell representation during training. Thereafter, a discriminator classifier is trained to ensure that predictions match the desired perturbation style. In other words, the IMPA model takes as input, a control image with information on a specific compound (e.g., topological and structural descriptors, perturbation) or a genetic perturbation. The information relevant to the drug or gene perturbation is used to guide the generating step and ensure that the generated image reflects the expected morphological changes caused by the perturbation. Qualitative analysis of IMPA predictions highlights the transformation of control cells into target perturbation while preserving content information such as cell orientation and translation. However, the training done with CP images is conducted with only five drugs, and was done only to demonstrate the feasibility of the model. The first assessment of IMPA qualitative inference consists of training a Random Forest classifier on extracted features (using CellProfiler) from generated perturbation images and original ones to discriminate control cells from perturbed cells. The results from the RF feature importance studies show similar shifts in morphological features for transformed images compared to controls.

Summary: The two proposed architectures open the door to phenotype-based drug discovery using CP data. IMPA’s architecture aids in predicting responses to unmeasured compounds by solely utilizing chemical representations for unseen drugs during testing. Zapata *et al.*‘s framework introduces novel chemical structures tailored to specific profiles, offering potential for generating hits in drug discovery without detailed knowledge about the target. Both papers illustrate how generative AI could navigate the perturbation space for optimal experimental design using morphological modification, coupled with gene expression.

## 4 Potential limitations and challenges of working with CP data

As stated throughout the review, CP assay data has a high potential for molecular activity or toxicity prediction. Compared to descriptors derived from molecular structure or also from, e.g., binding assays, morphological images or fingerprints give a more holistic view of a cell’s response to treatment, e.g., with a compound. In addition, the data can be generated in high throughput, making it extremely attractive for computational, especially deep learning, methods. However, the technique is still young and the real benefit for computational prediction depends on the understanding and the quality of the data sets. CP datasets comprise high-dimensional data extracted from single cells across one or multiple plates, often measured at different time points, which can yield to variability in the cell population. Handling protocols for sensitive cells may vary between laboratories, affecting cell response. The extensive information within CP datasets includes multiple replicates per treatment, resulting in multiple images captured by various channels. However, not all dimensions of this data are collected simultaneously, with replicates frequently distributed across multiple plates and taken at different time points. Thus, variability in experimental procedures can significantly affect cell populations and treatment responses, (Figures 1, 3). Thus, proper understanding of the experimental layout and adequate pre-processing, e.g., aggregation over cells and replicates, or corrections across plates of the data, are key.

No matter if available datasets are used on there own or combined, generating meaningful interpretations of the image-based data faces several challenges including variation between and within datasets. Observed data drifts, so called *batch effects*, mostly stem from experimental procedures and routines and, thus, must be considered - and ideally mitigated - during image processing and downstream analysis. The nature of the batch effects can vary widely, from generic environmental factors (e.g., humidity, temperature, assay location, experimentator) within the lab to factors that can be influenced to a certain extent before, during or after the experiments (e.g., handling, treatment, staining, and imaging of the cells, as well as data processing).

In the following, first the experimental and biological parameters that can lead to batch effects or other outliers will be discussed. Then, computational pre-processing strategies to account for these effects will be debated.

### 4.1 Batch effects before the assay has been performed

As the basis of cell-based *in vitro* screens, the integrity of the cells and standardization of their growth conditions is crucial for achieving comparable, robust results (Wang and Wang, 2022). Cancer cell lines, in particular, undergo genetic and transcriptional changes over time, leading to the diversification of the namely, same cell clone, and resulting in altered drug-responses (Ben-David et al., 2018). While it is possible to obtain authenticated cells from cell banks as starting material, the cell’s similarity can also be verified through sequencing. One factor contributing to this cell drift is changing cell culture conditions (e.g., cell confluency and reagents used within medium or for cell splitting) due to the lack of standardized cell culture procedures across labs. Accordingly, very detailed information on the culture of the cells should be provided with datasets and carefully examined. The cell lines chosen, as well as their growth conditions, should both align well with the research question at hand. Therefore, we strongly encourage the scientific community to apply CP to a broader diversity of cell types and growth conditions, such as fully confluent monolayers of epithelial cell lines.

Moreover, compounds to which cells are exposed are well-known to be prone to batch effects. Although this can only partly be influenced by scientist, compound quality and stability can be supported by optimal storage, handling, and solvent-compatibility of substances. With regards to systematical and non-biological batch effects, *in vitro* assays are prone to positioning effects (edge/drift effects), which can be mitigated with randomized compound layouts. Additionally, laboratories entering the field of CP screening may consider to initially screen a small set of common reference compounds, evaluating both positioning effects and reproducibility of in-house and published morphological profiles, following, e.g., HT screening or image-based high-content screening guidelines (Bray et al., 2004; Iversen et al., 2004), as well as CP reproducibility studies (Tromans-Coia et al., 2023). While negative controls are consistent across datasets, the inclusion of positive controls varies considerably across screens and is in need of harmonization. Positive control compounds ideally address both the MoA analyzed in the following screen and effect magnitude observed, while serving the purpose of the experimental set-up. However, this is only possible when investigating a specific MoA in a targeted screen. Nevertheless, even when CP is used as an untargeted screening method, positive controls can serve as important parameters to inspect systematic batch effects across screening sites or even within a dataset. We therefore strongly advocate for the joint definition of a set of approximately two to three positive control compounds that cover all stained cell structures (e.g., nocodazole and tetrandrine) and these control compounds should be included on every screening plate in large-scale screens using the same experimental set-up. An example of how to define and verify adequate control compounds was presented by Willis et al. (Willis et al., 2020).

### 4.2 Batch effects while performing the assay

Image-based screening methods assume that a few representative images from a well reflect the entire cell population. To ensure this, homogeneous exposure of the cells to chemicals during treatment is essential and must consider partitioning effects and diffusion kinetics within the medium. Adhering to recommended practices, such as those by Song et al. (Song et al., 2010), and integrating liquid handling systems in the procedure can support this. Nonetheless, comprehensive standard operating procedures for chemical and genetic perturbation of cells are still elusive and remain a challenge due to diverse technical equipment and protocols. In contrast, detailed descriptions of CP staining procedures are available (Bray et al., 2016; Cimini et al., 2023). Despite this, unintentional changes in cell seeding, growth rate, and staining concentrations are commonly experienced during screening between or even within laboratories, and have to be taken into account during data analysis. Notably, further optimization of the original protocol (Chandrasekaran et al., 2022; Cimini et al., 2023) and slight adaptations across screening sites may add further variation and should be considered when combining historic datasets with recently published datasets. For the imaging of treated and stained cells, technical equipment often differs between screening sites (i.e., microscope, operator, microscopic settings, and microscopic filters), introducing relevant batch effects that are difficult to avoid in the first place (Arevalo et al., 2023; Tromans-Coia et al., 2023). Interestingly, recent efforts identified relevant intentional choices in imaging settings (i.e., objective type, objective’s magnification, number and excitation sequence of channels, binning, number of z-planes, number of imaged fields and cells) that can contribute to variability between screening sides (Tromans-Coia et al., 2023). Authors observed an impact on data performance, especially by the number of available microscopic channels (four to six) and their excitation sequence (sequential/simultaneous) during imaging, as well as the number of cells imaged per condition. Importantly, the number of imaged cells strongly depends on the objective magnification and number of imaged fields per well, and therefore led to the general recommendation of using five fluorescent and the bright-field channel during imaging with a magnification of 20X and capturing approximately 2500 cells per well. Notably, researchers investigating morphological changes in RNA, ER, actin or Golgi structures, should pay attention to the selected channel separation options during imaging. This is important due to spectral cross-talk of dyes, which can influence the profile’s performance, and dye merging into one channel if less than six channels are available (Cimini et al., 2023; Tromans-Coia et al., 2023).

### 4.3 Pre-processing images obtained from CP assays

The process of object identification within images as well as their segmentation and feature extraction require homogeneous illumination to prevent artifacts introduced by microscopy optics or light sources. Although nearly all microscope systems used for CP perform an acquisition of variations in illumination prior to imaging of stained cells using an uniformly fluorescent reference sample, it is common practice to additionally perform a post-acquisition illumination correction to mitigate remaining illumination bias. This approach considers all or a sufficiently large subset of images and computationally improves lightning fluctuations in images of one plate and for each channel independently using correction functions (Bray et al., 2004). For this process, open-source (e.g., CellProfiler (Nyffeler et al., 2020; Willis et al., 2020; Way et al., 2021)) or proprietary analysis software provided with imaging systems (e.g., Harmony (Revvity)(Nyffeler et al., 2020; Willis et al., 2020)) can be used, which however rely on differing functions that are not always transparently communicated.

Chandrasekaran et al. (Chandrasekaran et al., 2023), Hofmarcher et al. (Hofmarcher et al., 2019), and Gabriel et al. (Gabriel et al., 2023) discuss various overlapping data processing steps. Foremost, high-resolution 16-bit TIF images are lowered to 8-bit PNG images, which mainly serves to compress the data, but can also serve to deal with image artefacts. For example, Gabriel et al. (Gabriel et al., 2023) normalized the 16-bit images to 8-bit images by rescaling the images from the 0th to 99th pixel intensity onto a 0–255 scale (i.e., 8-bit). This was done to remove outlier pixels with high intensities due to overexposure or other illumination effects. Normalization to the 8-bit range helps to further reduce variations attributable to image acquisition. Furthermore, the authors centre, crop and resize the images to a lower dimensionality. Hofmarcher et al. (Hofmarcher et al., 2019) describe a similar threshold for pixel intensity, and remove 0.0028%, presumably also the top percentile, of pixels with the highest values. Removing outliers and normalizing the data helps to standardize the image data. This helps to ensure that models later trained on these images learn from the actual content of the images, instead of artifacts introduced by the data collection process.

### 4.4 Pre-processing morphological fingerprints

**FIGURE 5:**
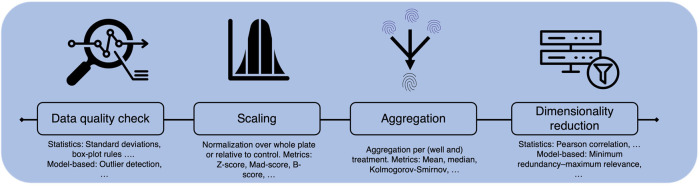
Flowchart illustrating morphological fingerprint pre-processing with metric examples, including quality control, outlier detection, scaling, and aggregation from single-cell to well to treatment levels, followed by dimensionality reduction for downstream analysis.

A standardised workflow for pre-processing data, before downstream analysis, typically involves quality data checks, scaling, and dimensionality reduction (Figure 5). However, the specificity of CP data requires an additional step: aggregation. Quality control of molecular fingerprints involves removing missing values and identifying outliers to eliminate unusual phenotypes. While this step needs to be performed in each study, there is often little information about the exact details and no specific protocol available to assure comparability.

#### 4.4.1 Dealing with outliers and different scales

Outliers may arise from experimental factors such as lab environment or manipulation errors as mentioned previously. However, unusual phenotypes can also result from dead cells. To identify these, one could examine the features related to the number of object (e.g., *number_of_object* where *object* can be *nuclei, cytoplasma, cells*). When the value of these features falls below a certain threshold relative to median value of the negative control (such as DMSO), it typically indicates dead cells. This step is a common step to reduce noise in the dataset. Statistical methods (standard deviation rules and box-plot rules, median and the median absolute deviation and Mahalanobis-based outlier detection) and model-based methods (supervised-machine-learning classifier) are also employed to remove outliers within the dataset. Despite the different methods available to identify outliers, this step is preferably performed on the whole population, considering all replicates without differentiation of plate, well, or treatment. Scaling involves adjusting data to a common scale facilitating statistical and computational operations. Various scaling metrics used with CP data include z-score (standardization used by Lapins and Spjuth (Lapins and Spjuth, 2019)) mad-score (robust standardization less sensitive to outliers as used by Pahl et al. (Pahl et al., 2023)), B-score (median polish-based algorithm notably used in (29)) and others (Bray et al., 2004). Once the metric is chosen, three normalization schemes are possible: 1) between plate normalization also known as normalization across all plates; 2) whole plate normalization, where normalization occurs within each plate, possible if samples are randomly scattered across the plate and if all plates have a similar proportion of active/inactive treatments; and 3) relative normalization, which normalizes all samples to controls or sham treatment depending of the number (preferably >16 /plate) and position across plate (preferably randomly scatters). As normalization across all plates is not recommended (Bray et al., 2004; Caicedo et al., 2017), the choice narrows down to the other two possibilities.

#### 4.4.2 Aggregation from single-cell to well to treatment

The morphological profiles extracted from images are at the single-cell level, meaning that a profile of features is extracted for each of the cells contained in a well. Population-level profiles (i.e., well level profiles) are obtained by aggregating these features into a single profile to facilitate population comparison and downstream analysis. Similarly, treatment-level profiles are obtained by aggregating all population-level profiles characterizing a treatment. Mean, median and Kolmogorov-Smirnov metrics are three commonly used strategies to create population- and/or treatment-level profiles. However, the median profile, which is less sensitive to outliers, is more often encountered (Caicedo et al., 2017). Finally, dimensionality reduction can occur either before or after single-cell aggregation. This process is intended to eliminate collinearity, gather or drop redundant correlated features, to decrease the number of features while retaining the most relevant and informative ones. Minimum redundancy–maximum relevance (Ding and Peng, 2005), support-vector-machine-based recursive-feature elimination,PCA and pearson correlation are metrics that can be employed.

### 4.5 Addressing CP data challenges with DL and other approaches

Despite CP assays and their use in DL being a relatively novel field without a universally solution for batch effects, there are options available. One can leverage noise mitigation tools developed for similar data types, such as microarray data (Chen et al., 2011). Recent work by Arevalo et al. (Arevalo et al., 2023) illustrates that computational batch correction methods developed for mRNA profiles can also mitigate batch effects in image-based data to some extent. However, their efficacy diminishes as scenarios become more challenging to align. Furthermore, validation of batch correction methods can be performed using gold-standard datasets, as demonstrated by Sypetkowski et al. (Sypetkowski et al., 2023). Moreover, the robustness of training contrastive learning models against batch effects can be enhanced by employing suitable learning strategies, as discussed in Section 3.2.3 by Gabriel et al. (Gabriel et al., 2023). They introduced two novel loss functions aiding contrastive learning approaches to disregard some variations in data attributed to batch effects.

It is crucial to acknowledge that there is no universal solution for handling batch effects. Thus, it is imperative to highlight potential batch effects in data through comprehensive metadata annotation. Furthermore, the identification of batch effects can be strengthened by incorporating a standardized set of compounds with diverse mechanisms of action in every batch of new CP assays. This facilitates batch comparison and correction across different assay settings. Establishing standardized preprocessing protocols and adopting a minimum information format to describe CP datasets is essential for enhancing reproducibility and making CP datasets more *FAIR* (Jacobsen et al., 2020). Therefore, both scientists performing the screens and bioinformaticians or computational chemists can contribute to improving the performance of models built on CP datasets in data across labs and studies when working closely together.

Machine learning models for risk assessment, trained on CP data, are showing promising results, though they are still in the early stages of proving their practical value. However, our analysis of various proof-of-concept studies suggests that CP data contains transferable information. This means that the results from one study can be used to train a better model to help predict the outcome of another study. We consider CP’s integration into the bio-activity screening pipeline valuable for risk assessment, yet its primary challenge remains to be effectively exploiting data aggregation. As more CP assays will be performed, and more CP data will become publicly available, ensuring effective data aggregation becomes increasingly important.

## 5 Conclusion

The integration of cell painting assays with computational methods shows encouraging promise for predicting compound activities and hazards in drug discovery and toxicology. By leveraging CP-based phenotypic data alongside structural information from compounds, machine learning and deep learning models can be developed to predict compound activities across various human-relevant disease endpoints and uncover underlying MoA. CP data offers potential advancements in comprehending compound responses within cells, thereby guiding therapeutic development and risk assessment while mitigating reliance on animal testing. It thereby provides complementary information to classically used descriptors such as molecular fingerprints. Although CP data provides undeniable information, such recent method comes with its sets of challenges and limitations. Addressing them calls for a collective effort to standardize protocols, promote reproducibility through the adoption of FAIR principles, and establish benchmark datasets for method evaluation. Additionally, efforts to improve understanding of image-based features and their biological interpretation are crucial. Identifying and prioritizing the most informative features will help optimise predictive models and enhance their interpretability. In summary, while CP offers significant potential for advancing compound evaluation and toxicology research, addressing the associated challenges is paramount to unleashing CP’s full benefits. By fostering collaborative work, advocating for standardisation, and improving pre-processing/methodological approaches, the scientific community could harness the power of CP data to drive innovation and advancement.
